# Community risk perceptions and recommendations for drowning prevention: A qualitative study across West Bengal, India

**DOI:** 10.1371/journal.pgph.0007377

**Published:** 2026-09-24

**Authors:** Debayanti Bhowmick, Soumyadeep Bhaumik, Jagnoor Jagnoor

**Affiliations:** 1 Injury Division, The George Institute for Global Health, Sydney, Australia; 2 School of Social Sciences, The University of New South Wales, Sydney, Australia; 3 Meta-research and Evidence Synthesis, Health System Science, The George Institute for Global Health, Sydney, Australia; 4 Injury Division, The George Institute for Global Health, New Delhi, India; Yale University School of Medicine, UNITED STATES OF AMERICA

## Abstract

Drowning is a leading cause of injury-related mortality worldwide, with disproportionate impacts on low- and middle-income countries. West Bengal, India, is characterized by extensive exposure to natural water bodies and marked regional and urban–rural heterogeneity. There is limited research on community risk perceptions and contextual drivers of drowning in this area, which constrains the design of effective prevention strategies. This qualitative study applied a phenomenological approach and conducted 46 focus group discussions involving a total of 339 participants from urban and rural communities across all 23 districts of West Bengal. Districts were selected based on recent drowning incidents. Adult residents were purposively sampled with support from local humanitarian organization. Data collection combined semi-structured interviews in local language, audio recording, and thematic analysis, iteratively coded to saturation using NVivo software. Four major themes emerged: (1) infrastructure and risk varies between urban and rural regions for instance differences in use and type of water sources, healthcare access and community connectedness; (2) Community awareness of population vulnerability, environmental risks, and other contextual exposures to drowning were identified where children, elderly, domestic migrant workers and non-swimmers were found to be vulnerable; (3) Adaptive response to threat through prevention, accessibility and rescue methods included informal rescue, reliance on traditional beliefs and expressions of fatalism; (4) Community recommendations based on lived experiences and risk perception of drowning were focused on fencing water bodies, enhancing childcare programs, strengthening medical infrastructure and providing swimming instruction. Infrastructure gaps and climate variability further heighten drowning risks in particularly exposed regions and households. Community risk perceptions underscore urgent priorities for drowning prevention in West Bengal. While findings are consistent across districts, intervention priorities vary according to regional needs.Strategies should strengthen supervision, physical barriers, childcare, water safety education, and access to critical care, informed by local perceptions and practices.

## 1. Background

Drowning is one of the leading causes of injury globally, with more than 300,000 fatalities reported in 2021 alone [1,2]. It is estimated that 92 per cent of total drowning cases occur in low-and middle-income countries (LMICs), and approximately 51 per cent of this burden is borne by China, Pakistan, Bangladesh and India [3]. Out of all drowning incidences, unintentional drowning is one of the 30 mutually exclusive injury-related mortalities in the Global Burden of Disease (GBD 2017) Study [4]. Thus, unintentional drowning forms one of the most important areas of inquiry when it comes to understanding the risk perception and formulating injury prevention strategies.

Unintentional drowning is accompanied by multiple predisposing factors like age, environmental factors, socio-economic conditions, cognitive characters and infrastructure [5]. As a result, the burden of drowning is not uniform across regions. Factors like geography, water proximity, livelihood patterns, and public exposure and age [1] tend to shape the distribution. Hence risk is not only associated with physical exposure but also how communities perceive water-related hazards and safety practices. Understanding these is critical, as they influence behaviour, preparedness, and the adoption of preventive measures.

Thus, prevention strategies for unintentional drowning call for regional estimation of burden and region-specific perceptions of drowning risk. Effective drowning prevention requires nuanced exploration of local perceptions and socio-cultural understandings of risk. This study seeks to contribute to this gap by examining how communities perceive drowning risk and prevention in relation to their lived environments.

This study focuses on the state of West Bengal in India with the aim of understanding risk perceptions on unintentional drowning across all its districts. With over 99 million inhabitants, West Bengal is the fourth-most populous state in India [6] with 23 districts. Topographically, the state has six regions, two of which are hilly and the other four being riverine, delta or coastal. In earlier studies, the Sundarbans region in the coastal south of the state was identified as one of the most disadvantaged areas in India with children facing a high risk of drowning [7,8]. However, our current understanding is limited to only the Sundarbans regions for children aged 1–9 years. The state, with its high population and exposure to ponds, rivers, coastal and delta regions, is likely to harbour more at-risk populations [9]. Thus, there remains a gap in our understanding of the drowning burden and risk perception among populations in other parts of the state that might reflect region-specific layers including plausible urban-rural differences.

This state-level qualitative study captures the region-specific factors that influence community perceptions of drowning. State-level data will broadly encompass the diverse topography of the region which can be linked to several drowning risks. Documenting community perceptions of drowning causes, at risk groups, prevention measures, and contextual understanding is crucial for developing implementation plans for known effective interventions and reducing drowning events.

## 2. Methods

A qualitative phenomenological method was applied to this study. Comprehensive Focus Group Discussions (FGD) were conducted to understand the risk perception of communities on unintentional drowning in West Bengal. Given the state’s vast geography and varied lived experiences, a phenomenological approach suited best as it is grounded in exploring experiences as understood by individuals living it [10]. This approach effectively captured subjective views and in-depth insights on unintentional drowning and associated factors shaping the risk perception and coping strategies in the community. It is similar to generating inductive themes, with the added acknowledgment that the perspectives produced are shaped by socio-economic and cultural conditioning, which in turn inform the embedded narratives surrounding the issue [11]. The study is reported as per the Standards for Reporting Qualitative Research [12] (See S1_SRQR_Checklist).

### 2.1. Ethics Statement

All participants provided written informed consent. The study was approved by the institutional ethics committee of The George Institute for Global Health (03/2024), Institutional Ethics Committee of Institute of Health and Family Welfare, Government of West Bengal, India (IHFW/IEC/7389), and the Health Ministry Screening Committee., Government of India (2024–19898).

### 2.2. Study Setting

The study covered all 23 districts of West Bengal. As per the first-ever waterbody census published by the Ministry of Jal Shakti [13], West Bengal accounts for the highest number of ponds, tanks and lakes (747,000) in all India. Of which 96.3 percent of the water bodies are located in rural areas and only 3.7percent in urban areas.

High exposure to open water bodies is one of the primary reasons behind the high burden of drowning in communities [9]. With increased disruption in weather patterns and water-related events, the risk of drowning is expected to rise if locally relevant interventions cross-sector surveillance are not put in place [14]. West Bengal, with its high number of open water bodies and extensive water zones, is exposed to high drowning risks [7].

The topographic diversity of West Bengal makes each district unique in terms of prevalence, risk exposure, and resilience. The study sites were also selected from both urban and rural areas to consider spatial stratification as a predisposing factor to drowning perception.

### 2.3. Sampling Strategy and Selection of Respondents

We conducted focus group discussions (FGDs) with adult respondents in all 23 districts of West Bengal in communities where there had been at least one drowning incident in the last 12 months. The recruitment was guided by the planned number of focus group discussions and the achievement of theoretical saturation. The study aimed to conduct at least two FGDs per district, with 6–15 participants per group. Based on the minimum anticipated group size of six participants across 46 FGDs, the minimum target sample was 276 participants. A total of 339 participants ultimately participated in the FGDs.

Overall, at least two FGDs were conducted in each district, until theoretical saturation was reached for the entire state with no new themes or insights emerging from the data. We conducted maximum variation purposive sampling (rural, urban) to identify the participants. This was done to capture common patterns cutting across a wide range of perspectives and experiences among the participants [15].

Information about the study was disseminated to potential respondents with the help of a locally based humanitarian organization- Child in Need Institute - through snowballing. Interested respondents were then explained the study details, and written informed consent was obtained.

### 2.4. Inclusion and exclusion criteria

Each focus group discussion comprised of respondents belonging to varied occupations. All respondents were adults aged 18 years or above and were living in the community for at least three years at the time of interview.

The exclusion criteria for participation involved those who were diagnosed with any cognitive/mental impairment and those not able to provide informed consent. This was to ensure that all participants were able to provide fully informed and voluntary consent.

### 2.5. Data collection

Data collection was conducted between August 22, 2024 and October 28, 2024, by three trained data collectors (Two females and one male). Recruitment of data collectors was based on qualitative study experience and research methods, local language proficiency and prior disciplinary qualification in relevant fields like public health and healthcare science. All FGDs were conducted face-to-face, and permission was secured to record each FGD session. A semi-structured topic guide was used (see S1_Text), which was iteratively revised as the study progressed. FGDs were conducted in the local language of Bengali. All the FGDs were audio recorded, and supplementary field notes were taken. Data collectors and field note takers conducted debrief meeting after each FGD. This ensured that key insights into the content of data were discussed for any changes in subsequent FGDs- thus strengthening the quality and trustworthiness of data in real time. An audit trail was maintained in excel sheets to record key decisions, to be traced and evaluated

### 2.6. Reflexivity

The authors comprised researchers with expertise in public health, epidemiology and social sciences. The research team included senior academics and early and mid-career researchers, bringing interdisciplinary perspectives to the study. All authors are currently based in Sydney and have roots in India.

To note, DB and SB are from West Bengal but do not have geographical or social lived experience in the specific study regions. SB has grown and lived in Burdwan (rural), Bankura (peri-urban) and Kolkata (urban) with an upper middle-income background. DB has grown and lived in Kolkata (urban) with a middle-income background. DB migrated to New Delhi, India in 2017 for higher studies and work. SB and DB have lived in India till 2022 and 2023, respectively.

All authors continue to have substantial links with different strata of society in Bengal through work, friendship, and education.

Although none of the authors possesses lived experience in the study region, all had prior experience working in rural and climate-affected communities, with JJ, as PI, having worked in the study area for over 11 years. This informed the study design and data collection process.

The authors engaged in ongoing reflexive practice**s**, including maintaining analytic memos while coding and going back to debrief notes from data collection. These were used to reflect on personal assumptions, positionality, and interactions with participants.

Regular team discussions were held to critically examine emerging interpretations and to minimise the influence of individual bias on coding and theme development. This process supported a more rigorous analysis of participants lived experiences. Transferability and dependability of the study is supported by contextual descriptions of the study setting and participants, and by following a systematic and transparent research process, including careful documentation of study limitations.

### 2.7. Data analysis

We transcribed all data verbatim through professional transcribers. This was cross-checked from audio tapes by researchers. For each focus group, a single researcher (DB) independently performed coding, starting with open coding on an initial set of five transcripts in the original language. Data collection and coding were conducted simultaneously facilitating constant comparison in an iterative and reflective manner for thematic analysis. The researcher then searched for conceptual relations between codes to develop concept maps, which served as the initial coding tree. These codes were data-driven, with more interpretive analysis emerging as coding progressed. This initial coding tree was applied to other transcripts as data arrived. As interviews progressed, the existing coding tree was modified iteratively. The final coding tree was then applied to all transcripts. Saturation was determined when no new themes or insights emerged from the data, indicating that additional data collection would not contribute further information. This was determined through debriefing notes which were maintained throughout the data collection process to document discussions and reflections on the data. The coding and final themes were reviewed and verified independently by other authors prior to finalisation.

NVIVO 11 (Version NVivo Pro) was used for all stages, except before the initial coding tree, which was done in hardcopy transcripts. No respondent back-checking was done due to logistical constraints.

The findings are supported by translated verbatim quotations from Bengali to English to provide contextual depth to the analysis. While maintaining participant anonymity, the location of each participant is reported at the end of the quoted excerpts. For participants from rural study sites, identifiers are presented using the village name followed by the district name. The village represent the smallest administrative unit in rural areas. For participants from urban study sites, identifiers are presented using the ward number followed by the district name, as wards constitute the smallest administrative subdivision within urban municipalities.

## 3. Results

### 3.1. Demographic Attributes

A total of 46 FGDs were conducted in this study across West Bengal, with each lasting between 30–45 minutes. A total of 35 FGDs were conducted in rural and 11 in urban communities. Overall, respondents ranged in age from 18 to 78 years, with women comprising the majority in most of the focus group discussions (See S1_Table). Respondents worked a variety of occupations such as home makers, construction workers, drivers, frontline workers, farmers, retired personnel and job goers. The heterogeneous pool of respondents reflected on a wide range of subjective experiences, risk perception, community-specific challenges, socio-economic conditions and critical takes on coping strategies and feedback on intervention policies.

The data was analysed thematically using an inductive approach. The transcripts were manually coded to derive initial themes and sub-themes. The analysis on risk perception is presented in the form of four broad themes presented in Table 1.

**Table 1:** Themes and sub-themes.

	Main Theme	Sub-Themes
Theme 1	Risk varies between urban and rural regions	• Usage and type of water sources • Choice of providers for post-drowning care
Theme 2	Community awareness of population vulnerability, environmental risks, and other contextual exposures to drowning	• Vulnerability across different age groups and conditions • Role of knowledge and familiarity in risk perception • Dependence on water for daily chores and livelihood • Cultural and religious practices around water bodies • Climate change and seasonal variability in drowning risk
Theme 3	Adaptive response to threat through prevention, accessibility and rescue methods	• Common rescue methods adopted by the community • Accessibility of formal healthcare
Theme 4	Community recommendations based on lived experiences and risk perception of drowning.	• Child-care interventions are the priority • Basic infrastructure improvements • Strengthening primary health care facility in the rural communities • Systemic access to affordable swimming lessons along with safety interventions around water bodies

While there was limited variance in themes identified between districts, significant differences emerged between rural and urban contexts, and discussed within the themes below.

### 3.2. Theme 1: Certain components of risk vary between urban and rural regions

Despite efforts to achieve a balanced representation of community types, drowning risks were comparatively higher in rural sites than in urban sites, likely reflecting the predominantly rural distribution of water bodies across West Bengal.

Nonetheless, there were noticeable overlaps in drowning narratives among all the communities. Perception did not vary greatly in terms of identifying the most vulnerable population, or priority intervention needs. Children were consistently identified as the primary victims. The causes of drowning recalled were similar, majorly linked to activities such as bathing in ponds, lack of supervision, or traditional practices.

However, it is important to note that these findings cannot be extrapolated to water-related occupations. Preliminary insights from our larger ongoing study evaluating the burden, context and perceptions towards drowning across West Bengal, India indicate that fisherfolk are also considered highly vulnerable to drowning risks.

Other key differences were identified in terms of usage of water sources, choice of health care providers and adhering to traditional beliefs.

#### 3.2.1. Usage of water sources.

In terms of usage, the urban communities acknowledged that water pipelines have reduced their reliance on natural sources for household chores. Thus, ponds are not perceived as an immediate risk factor for drowning.

*“Now every household has pipelines. Some have also invested to install submersible pumps. We don’t use the ponds that often”- (Ward 14, Kolkata-Urban)*

However, recreational bathing and religious engagements in water bodies remain common among the locals. As a result, risks among children, adults visiting for rituals and domestic migrant workers with lesser understanding of water risks continue to be a concern.

*“We visit ponds during festivals or rituals. I wouldn’t say we don’t use ponds at all. We do use them during special occasions…Many Brahmin households also use the water for daily religious purposes or just to take a dip.” - (Ward 2, Bankura - Urban)*

In contrast, rural communities were considered more at risk compared to their urban counterparts. This is due to the nature of their livelihoods in agriculture or fishing, use of open water bodies for daily chores, and the topography of the area, which bring them into closer proximity to natural water sources as part of their daily routines,increasing the risk of drowning. Experiences recalled by rural communities were much more nuanced. Given their higher exposure to water through everyday living, drowning risks were more frequent and feared.

*“We use pond water to wash the utensils and clothes. We do have a tap at home, but we just use it for storing drinking water. The water is neither frequent nor adequate. We have no choice but to depend on pond water.” – (Raghunathpur, Purulia- Rural)*

They believed that the risk could be mitigated through expanded access to piped water systems, provisioning of infrastructure beyond fencing and coping with the changing dynamics of water bodies and climatic factors.

#### 3.2.2. Choice of providers for post-drowning care.

Another notable difference identified was in the choice of providers for post-drowning care. Majority of respondents from urban areas preferred formal healthcare providers, as they had access to essential medical facilities and specialized services.

*“The Hospital is near. We have the health centre. There are doctors near the Municipality too. There are three doctors” – (Dhupguri, Jalpaiguri- Urban)*

However, a few communities also mentioned the lack of local healthcare options, leading to reliance on informal doctors/quacks, though such accounts were fewer in comparison to rural communities.

In rural areas, access to formal medical care was limited, prompting many to turn to quacks or traditional healing methods like spinning the victim on their heads. This is driven by distance, cost, and weak infrastructure.

*“The government hospital is very far. Not nearby. No hospital near Lalgola. We have small-scale doctors around. We go to them first.” - (Mannantali, North Dinajpur- Rural)*

Lastly, urban regions experienced a marked sense of community disconnection. Respondents in these areas often displayed a lack of engagement with each other, which contributed to a general unawareness of drowning incidents occurring nearby. Many communities were unable to recall recent drowning cases in their vicinity.

### 3.3. Theme 2: Community awareness of population vulnerability, environmental risks, and other contextual exposures to drowning

#### 3.3.1. Vulnerability across different age groups and conditions.

In almost every community surveyed, children up to 10 years and adolescents were identified as the most vulnerable age-group who are exposed to drowning. With exposure to numerous community ponds and water bodies, children lack awareness of potential danger and cautious behavioural skills. The next high-risk groups identified are youth falling in the 20–25 age bracket and elderly population. We assume that references to ‘elderly’ individuals were intended to denote older adults aged 60 years and above, consistent with commonly adopted definitions in the Government of India’s *National Policy on Older Persons* [16].

The causes of drowning are intricately associated with the at-risk population. In the case of children, lack of supervision has been identified as one of the most common causes of drowning by the community. Most children are fond of water.

*“We do not use the pond much. However, my son is very fond of it… he will always run towards it whenever he gets a chance”- (Brajalalchak, Purba Medinipore- Rural)*

With parents engaged in livelihood and household chores, it is often difficult for elders to always supervise the child’s whereabouts. In this context, one of the respondents mentioned:

*“Such incidents happen with children when mothers are busy elsewhere, for example they went to fetch water or maybe cooking meals. It is then that such accidents happen. If we engage the child in some games or have anybody else who can supervise them, it will help us prevent the mishaps.” - (Kakjol, S24 Parganas- Rural).*

For elderly population and persons with cardiovascular and mental conditions, safely accessing water bodies is important. Lack of fencing and risk prone water zones (like open ponds or bodies where environmental conditions increase the risk of drowning) are added dangers especially with the slightest lapse in caution. Moreover, individuals in an inebriated state are particularly susceptible to these hazardous areas.

#### 3.3.2. Role of knowledge and familiarity in risk perception.

It is also observed by many respondents that domestic migrant workers or tourists belonging to different regions are more prone to drowning than the local inhabitants.

*“The ones who have drowned, none of them are locals, they are non-bengalis and migrants”, - (Panihati, North 24 Parganas- Rural)*

Inter and intra-state domestic work migrants and visitors engaging in recreational swimming or religious travel lack knowledge and understanding of the waterbody that they are accessing. Lack of knowledge among the non-swimmers also proves fatal when they are unable to assess risk and sudden climatological factors.

*“For instance, I once visited Ganga and didn’t know that the zone was risky. As soon as I got into the water the locals pulled me out saying it’s the time for high tide and the water may rise anytime” – (Ward14, Kolkata- Urban)*

Many respondents have noted that the increasing disconnect from water bodies has negatively affected the coping skills of children in the recent generation, who possess little knowledge of water safety and survival techniques.

*“Earlier days, we were more dependent on the water bodies around us. We accessed the pond for every household chore. But now we do not visit ponds that often. A lot of us don’t know how to swim” - (Gobaranda, Purulia- Rural)*

#### 3.3.3. Dependence on water for daily chores and livelihood.

With a high proportion of households lacking access to piped water, reliance on natural water sources becomes essential for daily chores and livelihood. This dependency inherently raises exposure to water bodies, which in turn increases the risk of drowning. A substantial proportion of the rural households are dependent on natural sources for their household chores and hygiene requirements. Even in households where pipelines have been installed, water is inadequate to fulfil requirements in large-sized households.

*“Many of us use the water to make rice...some clean their utensils...some wash clothes. The water from tubewell isn’t very nice for cooking. Some put ‘alum’[translation: aluminium sulphate] to make it more drinkable”- (Enatnagar, Murshidabad- Rural)*

Other than the daily household needs, water is also an important source for livelihood in areas which are fishing intensive.

*“Every pond in the village has become unclean. Most of them are being used for fishing..sillage is put in the water..these ponds are no more used for anything else”- (Dangsara, Purba Barddhaman- Rural)*

#### 3.3.4. Cultural and Traditional practices around water bodies.

Several traditional practices in India tend to take place around water bodies. Annual festivals, traditional rituals and daily religious activities often involve natural water sources as the primary location of interest for the purpose of worshipping. A high number of cases have thus been attributed to risky exposure to water bodies during similar traditional practices.

*“A few days back, he went to the Ghat where people go during Makar-sankranti festival. The bank was full that day. He went a little deeper from the usual safe zone to take a dip away from the crowd. He couldn’t fathom the risk” - (Biswanathpur, Jhargram- Rural)*

It is reported that while some mishaps could be avoided by putting safety measures in place, some couldn’t be prevented.

We also see a stark urban-rural difference in such practices. Although festivals and traditional practices surrounding water are common among urban-Hindus, fewer people engage in specific rituals related to drowning emergencies. The large-scale and seasonal rituals are widely followed, while regular, small-scale rituals are not. Hence, risk exposure with respect to traditional practices around water bodies are comparatively lesser in urban than rural areas.

*“No traditional practices as such. Yes, we do pray to them when any mishap happens. But we Bengali Brahmins don’t believe in any specific ritual or ceremony where one can be cured. Though I am not sure about any other community” (Ward 2, Bankura- Urban)*

Nonetheless, few elderly respondents in urban areas were likely to uphold traditional practices in response to a drowning incident, which is often associated with deep-rooted cultural connection. Contrastingly, in rural areas, ponds and rivers were often seen as an integral part of communities’ belief system, with worshipping water sources after a mishap or organising a memorial service following a death being common.

*“We call it ‘Janma Pujo’ i.e. worshiping the water god. We do it because the incident happened there. The Pujo gives us peace, helps us cope” – (Raghunathpur, Purulia- Rural)*

#### 3.3.5. Climate change and seasonal variability in drowning risk.

The perception of risk prevalence was also linked to seasonal and climatic factors. Increased frequency of cyclones and storms have induced higher levels of flood especially in low-lying deltas of the state.

*“Once in every year, the storm floods the whole village. The whole region remains submerged under water. The reason why monsoon season could be difficult” - (Ward 91, Paschim Barddhaman- Urban)*

Respondents in flood-prone regions identify the rainy season as a critical time for drowning risks. Increased flooding and storms elevate water levels in both rivers and ponds, making it difficult for households that depend on these water sources for daily living to cope with the sudden climate-related stress, often leading to drowning incidents.

*“Respondent 1: It’s always risky during monsoon. Yet we need to go and bathe...we need to go for other chores. We can’t afford to live in fear -(Basuria, South Dinajpur- Rural)*

*Respondent 2: When the region floods, rainwater fills the fields, and the nearby river swells to its brink. As the water level rise beyond the limit, the village eventually gets submerged”- (Basuria, South Dinajpur- Rural)*

In rivers, the risk is also tied to the unpredictability of water flow and the impact of incoming weather patterns, which can make the water turbulent and challenging to navigate. With water flooding the roads, infrastructure in pond and river-exposed communities often fail to mitigate rising water levels. Sudden storm surges and high tides also make rivers risky during the day or monsoon season. Lack of electricity and proper roadways aggravate the precarity of the situation, limiting access to health services.

Conversely, some respondents view warm weather as a key factor drawing people to water bodies for recreational bathing and swimming. Households that rely on water bodies for leisure activities were particularly attuned to this risk.

*“Risk is higher during the warmer months when people frequently visit the water sources to take a dip. These mishaps happen then. During monsoon, people are too scared to go near the high-water levels”- (Matigarah, Darjeeling- Rural)*

### 3.4. Theme 3: Adaptive response to drowning through rescue methods, accessibility and prevention

#### 3.4.1. Common rescue methods adopted by the community.

The responders shared some common rescue methods used by the community. Due to the risk of drowning, people often stay close to the water to monitor and look out for anyone who is swimming or in the water. They make sure children stay away from the pond, supervise each other’s children, and collectively monitor high risk areas.

*“We are always looking out for each other, someone or the other is always around when they notice children nearing any pond. They scold them and tell them to return home. The reason why children returns back home”- (Eruar, Purba Barddhaman- Rural)*

If someone is drowning, they quickly try to help by reaching out with a strong tree branch or bamboo, calling for help, and informing authorities such as the police and nearby army cantonment (if any) to send a rescue team. Sometimes, swimmers jump in to help, but many in the community know even skilled swimmers can be at risk, so they avoid dangerous rescue actions that might make the situation worse.

*“We will try to save them, but we must ensure our safety too. We can’t help if we drown ourselves. The reason why we need to find an ideal ‘position’ and technique to save the victim from drowning. We must then try and press the water out if possible” - (Gobaranda, Purulia- Rural)*

Though most urban communities are well versed with medically approved resuscitation methods, in many rural regions, the most practised rescue technique involves spinning the victim on the head until they spit the water out. Though not considered a medically approved technique, many success stories made the communities adhere to it. It is also very common among certain rural communities to resort to traditional customary practices such as worshipping near the water source or to some deity either as a preventive measure against drowning or immediately after a rescue. This is aimed to stop further drowning incidents or to prevent death or pray good health of a surviving victim.

*“After the victim is rescued, we lie them down…and then we pick them up on our head and spin…spin for as long as 2-3 hours. We believe that no person immediately dies after drowning. They stay alive for the next 2-3 hours and the spin will help them survive. We also beat the trees around us, we believe the soul must be trapped there.” – (Ward 10, Hooghly- Urban)*

Respondents were also concerned that finding a doctor would take too much time and was one of the reasons behind resorting to these practices. Not being aware of the correct resuscitation methods makes them rely on this traditional approach.

Another very prominent response in both urban and rural communities is the expression of a rigid form of fatalism when discussing the drowning incidents.

*“The one who dies had to die. Their fate is already written. Nothing can stop that. We can’t do anything about it.” – (Ward 91, Paschim Barddhaman- Urban)*

The underlying belief is that the victims were somehow destined to suffer such a fate, and that the tragic outcome was inevitable. This perception is associated with delayed intervention or no intervention at all, given the presumption. Incidents may also go unreported because of the belief that circumstances are unavoidable.

#### 3.4.2. Accessibility of formal healthcare.

Drowning injury calls for immediate medical attention. The time to access emergency care or proper medical attention plays a determining role in ensuring quick mitigation. Most of the communities rely on the nearest health centre for quick medical attention. Communities belonging to urban counterparts largely rely on nearby private or public super-specialty hospitals as connectivity isn’t a major challenge. In rural regions, Primary Health Centres (PHCs) often serve as the last resort for medical referrals due to the shortage of healthcare personnel and limited availability of alternative facilities.

*“Respondent 1: He [the informal doctor] visits when anybody’s ill…prescribes medicines and saline when required. We don’t consider him a quack doctor, he’s a compounder, works at the pharmacy.*

*Interviewer: Do you get health services at the nearest health centre?*

*Respondent 2: Not much. The Primary Health centre operates two days in a week. If something happens in the remaining five days, they will just prescribe minimum medicine as there would be no doctors for a detailed check-up. For emergencies and complications, we have to visit Mahakuma Hospital or Barddhaman District hospital, far from here” – (Eruar, Purba Barddhaman- Rural)*

Households with access to personal vehicles can afford to commute longer routes to access higher level facilities. However, in case of emergencies, faraway centres may not be a viable option due to the time-sensitive nature of critical care post-drowning. Under such circumstances, seeking care from informal practitioners is the immediate response. Quack doctors and traditional healers have been an integral part of the rural health system catering to sections where accessibility has been the foremost issue.

*“Respondent: We take them to the traditional healer and sometimes to the hospital.*

*Interviewer: How far is the hospital?*

*Respondent: 9 KM from here...more than that I think...three extra from the village.. that would be 12 KM in total. It takes more than 30 minutes to reach. These days we take the ‘toto’ (electric rickshaw) to get to the hospital. Theres is some problem with charging the vehicle. But everyone with toto access prefers going to the hospital” – (Basuria, South Dinajpur- Rural)*

### 3.5. Theme 4: Community recommendations based on subjective experiences and larger risks of drowning

#### 3.5.1. Child Care interventions are the priority.

Increased vigilance around children, including stricter supervision, is a primary method adults use to ensure that children are not exposed to risky water zones. Some also tend to avoid exposure to water bodies completely.

*“Respondent 1: We are always very cautious. For instance, I tend to stay away from water because I don’t know how to swim. I don’t take a bath if the electricity is out. For me, the pond or river is never an option.*

*Respondent 2: Parents are also alert. Since the ponds are near, there is always someone to supervise the child. But mistakes may happen, one might get a little distracted while their children wander off, or cases involving people with physiological conditions. Things go out of hand only then” – (Islamabad Birpara, Alipurduar)*

With children and teenagers perceived as the most vulnerable population to drowning risks, most of the recommendations focused on ensuring their safety and enhancing childcare provisions. Fencing ponds and putting barricades along riverbanks were the most frequently suggested measures to prevent drowning incidents.

*“If the pond could be fenced and locked. Open the fence only during use and then lock it later. Children would never.”- (Bargodagodar, Purba Medinipore- Rural)*

*“The government can build a barricade along the riverbank. The possibility of accidents will massively reduce. This could be done also on the riverbed to limit the access to risk zones. They make those during festivals where access is granted only up to a safe zone. The rest is demarcated as danger” – (Mannantali, North Dinajpur- Rural)*

Additionally, respondents emphasized the need for a reliable daycare system. With parents facing challenges in providing strict supervision, daycare centres were seen as a vital resource for caring for children when parents are engaged in work.

*“Respondent 1: If we had any facility where mothers can admit their children where they could be looked after for the day*

*Respondents 2: The Integrated Child Development Services (ICDS) position centres are good avenue for that, but they shut down at 10 AM and there is nobody who can look after the children. Moreover, it is not possible to keep Children under 3 y/o at the ICDS. We need separate facilities for infants”- (Dangsara, Purba Barddhaman- Rural)*

In a similar context, one mother suggested the use of enclosed playpens as an efficient solution to ensure closer supervision of young children.

#### 3.5.2. Basic infrastructure improvements.

The communities mentioned how dependency on water bodies for household chores and hygiene is an integral part of their daily life. In a similar rural context, some pointed out that exposure to water bodies is higher where pipelines and access to safe drinking water is a challenge.

*“The government has installed a tap in the household. But the supply isn’t regular. The water is also full of iron. This is the main problem. Under such circumstances if there’s a river nearby, people will definitely go there, and eventually put themselves at risk” –(Chartkatala, Nadia- Rural)*

*“Respondent 1: Before everything else, we believe availability of water should be considered as the highest priority at the moment. That would help reduce the usage of pond.*

*Respondent 2: If there’s a tap in the house, children will no longer follow the mothers to the pond”- (Sitarampur, South 24 Parganas- Rural)*

Lack of a proper pipeline and drainage system was identified as a major infrastructural lapse, directly associated to drowning risks. In contrast, communities with pipelines installed did not perceive drowning to be a frequent risk, largely due to reduced access to water bodies.

Roadways, connectivity, and electrification were also identified as critical infrastructural issues that require immediate attention and improvements for safer commute and adaptation.

*“The roads must be levelled and made higher than the ponds. If not, children often fall when the roads are flooded” – (Daspur, Paschim Medinipore- Rural)*

*“There was a non-fatal drowning here. A three-year-old child fell into a pothole, which got filled with water during rain”- (Harishpara, Malda- Rural)*

#### 3.5.3. Improvement of PHCs.

A responsive primary health care facility is a pressing need felt in rural communities. Delayed care is often cited as the primary cause behind drowning fatalities, whether due to unreliable primary health centres (PHCs) or long travel times to the nearest emergency services.

*“The nearest hospital is 14 KM away. It’s more challenging if there’s any emergency at night. It’s also difficult to rent a car here. We are living very close to the forest, without a car, it gets difficult to commute, especially due to the elephants here. We do not even have a pharmacy in this village”- (Chotokendubari, Jhargram- Rural)*

Improving healthcare staff, availability of beds, and improving equipment may encourage rural victims to use PHCs as their first points of contact.

*“It will be very convenient if there’s a doctor in the health centre, then we wouldn’t be commuting as far as Birpara or Madirhat, but visiting the centre instead. Also, if arrangements are made to keep the required First Aids and other essential resources. The public will greatly benefit from this” – (Islamabad, Alipurduar- Rural)*

Public health interventions came up as an important community recommendation to tackle drowning problem. This included the need for robust primary health care facilities, with prompt emergency and other curative services.

#### 3.5.4. Systemic access to affordable swimming lessons along with safety interventions around water bodies.

Swimming recurred as one of the most important preventive strategies for both adults and children, in drowning prevention. Complete lack of exposure to water bodies has been interpreted as a negative way of life where children are less equipped to deal with water-related accidents. As a coping skill, children are taught swimming under the supervision of qualified instructors or experienced adults to help them understand the water bodies around them and adapt to risky water.

*“We have started to distance ourselves with water. The ones who do not go to the ponds usually take swimming lessons at pools. We must learn the technique without exposing ourselves to risks” – (Gobaranda, Purulia- Rural)*

Learning to swim equips individuals with vital skills that enable them to stay afloat, understand the physiological dynamics of the natural body and to cope with possible emergencies. Adhering to safety protocols around water bodies was seen not only as a matter of individual responsibility but also as a need for systemic interventions that the community considered essential. The most common recommendation was provisions for affordable swimming lessons and installation of warning signs and presence of rescue teams at busy riverbanks.

*“I believe in swimming lessons. We are always busy with work… can’t give much time or teach while we work. If somebody else, does it for us it will be helpful. Someone who would take and drop the child safely, pre and post session”- (Sukhdebpur, South Dinajpur- Rural).*

Additionally, the availability of rescue boats, life jackets, and rescue tubes was frequently mentioned as critical measures for improving safety.

While public swimming pools are an emerging option in urban setting, ponds and rivers have historically provided an affordable and viable option among rural communities to learn swimming. However, due to the recent widespread degradation of ponds caused by unsustainable fish farming, pollution, and the frequent digging of waterbeds, many of the natural water sources have become unsafe and unusable.

*“We have places to learn swimming, but they aren’t clean. The pond is full of algae. You can see how filthy it is right now. All households dump their garbage in it. Earlier when population was little, the water used to be clean. During flood, commute becomes difficult especially to reach the hospital” – (Dhupguri, Jalpaiguri- Rural)*

As a result, a key recommendation has focused on the restoration of ponds and rivers, with an emphasis on making them cleaner, safer, and more suitable for swimming activities. One of the most criticized practices has been the unethical excavation of pond and riverbeds for free mud and sand which has resulted in deeper, non-standardized water depths.

*“They need to stop digging the riverbeds for soil. This would help prevent accidents”- (Mekhliganj, Coochbehar- Rural)*

This makes the water more hazardous, particularly for children, increasing the risk of accidents.

## 4. Discussion

The findings of the study present community risk perceptions of drowning in West Bengal, India. It is observed that the presence of water bodies plays a crucial role in shaping the perception and knowledge within communities. Their position, presence of open water sources, daily interactions, and cultural associations all contribute to how communities perceive and manage drowning risks. This can be traced to similar knowledge systems observed among several communities across the globe. For instance, in the Maori community in New Zealand, traditional Maori knowledge, values, and ethics shape their customary practices for freshwater monitoring and management [17]; Stewart‐ [18]. Similarly, many Aboriginal cultures in Australia recognize water as a defining element for language boundaries and ceremonial sites, and it underpins various land management practices [19,20]. This study underscores how water-related knowledge, survival skills, and prevention strategies often led by the community elders stem from local knowledge systems, local traditions, and close human-environment relationships.

The study shows that the community well identifies the vulnerable population, the different factors affecting drowning and their exposure and response to the risk. Drowning has been identified as a pressing concern among children which aligns with earlier studies from West Bengal, where Lukaszyk et al. [21] highlighted the vulnerability of similar kind, and Gupta et al [22] reported mortality rates of 243.8 per 100,000 children aged 1–4 years and 38.8 per 100,000 children aged 5–9 years. The findings in this study suggest that there is also a prominent risk among other vulnerable demographics such as young adults (20–25 years), the elderly (60 and above), people with physiological and cognitive conditions, tourists, domestic migrant workers, non-swimmers and people under intoxication. It is recognised as a medical emergency and respondents identified a multitude of factors that made coping difficult including infrastructural barriers, climatic factors and changing water dynamics. On the public health front, we found resource availability, distance and traditional belief systems, influencing healthcare choices. We also identified marginal differences in certain component of risk between urban and rural regions, primarily determined by varied usage of natural water resources, choice of healthcare providers and traditional belief systems. In terms of prevalence, all respondents could relate to varying seasonal prevalences. In some areas, warmer weather conditions were seen as a key factor, drawing more people to water bodies. Conversely, flood-prone regions identified monsoon as a critical time for drowning risks. Overall, the relative consistency of findings relating to risk perceptions, vulnerability, and adaptation needs across 23 geographically diverse districts is noteworthy. This commonality is an important finding in itself, suggesting that the issues identified may reflect a broader state-wide pattern. The diverse contexts support the transferability of the findings and suggests that the patterns are not localised phenomena but reflect challenges experienced across multiple regions of the State.The respondents identified an array of recommendations based on their lived experiences and feasibility Like prioritising childcare intervention, infrastructural improvement including primary health care and increased access to affordable swimming lessons.

Previous perception studies on drowning in Bangladesh [23] and South India [24] specifically explored drowning among children. This study is set within a sub-national context and recruited community level respondents, enabling us to look at the issue in greater detail, and for the West Bengal context. Conducting a state-level analysis also presents us a broader perspective, shifts the lens to other demographics and puts together a subjective narration of experience. The failure of the community to translate their knowledge into intervention was one of the key findings in previous work [23]. Our study on the other hand highlights detailed community responses and strategic techniques applied to cope with drowning risks. The community presents us with multiple intervention strategies which are perceived to be of highest priority. Recommendations to strengthen infrastructure, primary and preventive health care systems, restoration of water sources and putting safety protocols in place to address unintentional drowning injury. The finding that lack of supervision still plays a major role in relation to drowning among children is consistent with what has been seen in other studies on drowning risks among children [25–28].

Time and again, drowning has been considered a critical public health concern [29,30]. This study is a documentation of community understanding of factors affecting drowning and their responses. Literature on drowning prevention and intervention strategies [31–34] often links drowning to socio-behavioural attributes and educational themes. Our study affirms to that and additionally presents a state overview of multi-factorial nature of the threat.

The themes identify community awareness of risks, adaptive responses to the threat and recommendations based on experiences. Used prospectively, the findings can help design effective interventions and improve programme compliance, addressing the issue highlighted by Rahman et al. [23] about the gap between community knowledge and action. For instance, the study by Gupta et al. [7] identified specific intervention adaptations appropriate to the local context of the Sundarbans region in West Bengal. One key finding was that preferences for barrier-based interventions varied across households, leading to the suggestion that customised barriers should be delivered to meet individual household needs. The study results are consistent with this finding, suggesting that interventions need to be locally contextualised according to household experiences and needs. Saturation was attained at the state level for each theme. We acknowledge the limitation that higher proportion of women participation in the FGDs affected the gendered nuances of risk perceptions to drowning in the communities. Given the high burden of drowning among children, women as primary caregivers, tend to respond more in the line of supervision role, risk perception and preventive practices for children in the community. Though the insights majorly covered child-specific vulnerabilities and community-level safety practices, consistent probing also helped us generate insights beyond this. These insights present the perspectives of the community and are part of their everyday experiences in general. With the research team currently undertaking parallel analyses focusing specifically on fisherfolk, boatmen and tourist-related drowning risks, we observed that responses and perceptions regarding high-risk populations in specific settings, compared with those of the broader community, generate a range of other community-based perspectives with notable overlaps.

Nonetheless, we acknowledge that this composition may have influenced the scope of the findings by underrepresenting male-specific exposures and behaviours that are often associated with higher drowning risk within specific livelihood settings.

Secondly, the study draws on verbatim translations from Bengali to reflect local community perceptions. However, these perceptions are often generalised in nature. Consequently, vulnerability is presented in broad terms and may not capture nuanced, medically or demographically disaggregated categories such as specific age groups or health conditions.

Lastly, it is difficult to conclusively determine whether the low incidence of drowning in urban settings reflects low occurrence, awareness or underreporting. Drawing on qualitative insights, we suggest that this pattern is more likely influenced by social disconnectedness in urban settings, where collective awareness is weaker than in more cohesive rural communities.

Nonetheless, we ensured that respondents had a wide age-range and belonged to diverse occupational and locational backgrounds to capture a wider spectrum of perspectives.

It is envisaged that the findings will enable policy makers and implementors to identify additional areas of intervention.

## 5. Conclusion

State level analysis of community risk perception can capture variations in topographic and demographic characteristics across different regions. Emerging themes addressing threat appraisal, adaptive capacity and recommendations become helpful while dealing with structural gaps and factors which facilitates the dangers of drowning. Community-based recommendations, along with region-specific needs, are crucial for communities, practitioners and policymakers to design effective and contextually appropriate interventions.

## Supporting information

S1 SRQR Checklist Compliance Checklist of Reporting Qualitative Research Standards. (PDF)

S1 Text Drowning in communities - Topic guide for FGD. (PDF)

S1 Table District-wise Demographic Characteristics of Respondents. (PDF)
