# Supplementary material for: Community risk perceptions and recommendations for drowning prevention: A qualitative study across West Bengal, India
Source: PLOS Glob Public Health. 2026 Sep 24;6(9):e0007377. doi: 10.1371/journal.pgph.0007377 (PMC13606942; doi:10.1371/journal.pgph.0007377)
Supplement: Supplementary file 1 [file pgph.0007377.s001.pdf]

S1 SRQR Checklist: Compliance Checklist of Standards for Reporting Qualitative Research (O'Brien et al., 2014)

Title & Abstract

- [x] Title identifies qualitative nature, approach, or method
- [x] Abstract summarizes background, purpose, methods, results, and conclusions

Introduction

- [x] Problem formulation clearly described and supported by theory/empirical work
- [x] Purpose explicitly stated

Methods

- [x] Research paradigm identified (constructivist)
- [x] Researcher characteristics described
- [x] Context and setting explained
- [x] Sampling strategy detailed with rationale
- [x] Ethical issues addressed
- [x] Data collection methods explained
- [x] Data collection instruments described (guides, tools, development process)
- [x] Units of study (participants) specified
- [x] Data processing procedures explained
- [x] Data analysis methods described
- [x] Techniques to enhance trustworthiness (Debriefing)

Results/Findings

- [x] Synthesis and interpretation of data presented
- [x] Empirical data (quotes) provided to support findings

Discussion

- [x] Integration with prior work and literature
- [x] Implications discussed
- [x] Limitations acknowledged

Other

- [x] Conflicts of interest disclosed
- [x] Funding sources reported
