# Supplementary material for: Community risk perceptions and recommendations for drowning prevention: A qualitative study across West Bengal, India
Source: PLOS Glob Public Health. 2026 Sep 24;6(9):e0007377. doi: 10.1371/journal.pgph.0007377 (PMC13606942; doi:10.1371/journal.pgph.0007377)
Supplement: Supplementary file 2 [file pgph.0007377.s002.pdf]

S1 Text: Drowning in communities - Topic guide for FGD

Interview Details

Participant ID: DIST/XX

Researcher Initials |__|__|__|

Note taker initials |__|__|__|

Location of interview:

Date |__|__/__|__/__|__|

Introduction

Thank you for agreeing to give the interview. I appreciate your willingness to participate and the time you have taken out of your day to take part. I am ______________________________ from ______________________ and the ___________________, my colleague is the note-taker. We are conducting these interviews in to understand your views and experiences in relation to drowning in in West Bengal, India. This will help us address drowning in the state.

- Explain consent.
- Give opportunities to ask for clarification.
- Ensure written informed consent is taken and participant information sheet copy is given to participant.

Setting ground rules

First, I would like to cover some general rules about how this discussion will run:

1. We would like you all to do the talking and to hear from everyone in the group.
2. There are no right or wrong answers – every person's opinion and view is important, and we would like to hear a range of different views.
3. Only one person should speak at a time so please wait for the person talking to finish.
4. We will be recording these discussions because we want to capture everything you have to say accurately. But please be assured that we do not identify anyone by name in any report and your identity will remain anonymous.
5. Does anyone have any questions?

Topic Guide

Domain	Topics
**Starting off**	Please tell us a bit about yourself, and your community?
**Perceptions of drowning**	• What are your thoughts and experiences regarding drowning in your community?<br>Probe and Prompt<br>- Magnitude and change in magnitude in past years<br>- reasons for magnitude of burden (high or low burden both should be asked)<br>- Attack by wild in water

	- Ability to prevent.<br>- Climate change<br>- Any intentional causes<br>• You have had drowning incident in your community Please describe the situation. What happened? How and when did this happen?<br>Probe<br>- Were you or anyone around when this happened? If yes, what did you/that person do to help?<br>- How do you think this could have been prevented?
**Drowning risk**	• Who gets affected by drowning or prone to the risks of drowning?<br>Probe (also ask Why)<br>- Male/ Female<br>- Difference between boys and girls / men and women Why?<br>- Children<br>- Adolescents<br>- Elderly people<br>- Any special population<br>• When are people more vulnerable to drowning?<br>Probe<br>- Time of year / season<br>- Specific event (festival or extreme weather event)<br>- Any behaviours and activities<br>- Swimming skills
**Health seeking behaviour for drowning**	• If you saw someone drowning, what would you do?<br>Probe<br>o Personal measures<br>o Would you try to save them yourself? Why is that?<br>o Swimming skills<br>• What would you do after the drowning incident has occurred?<br>Probe<br>o Personal measures<br>o Personal Practices/ rituals<br>o Would you seek care? Where and who would you seek care from?<br>o first aid center<br>o doctor<br>o PHC<br>o District Hospital / Medical college<br>o local pharmacist<br>o traditional healer<br>o Why would you seek care from this particular person/facility? ?
**Current safety practices**<br><br>**Suggestions for future**	• What activities or measures are you aware of in your community that are being taken to prevent/reduce drowning?<br>• What activities or measures are you aware of in your community that can be taken to prevent/reduce drowning?<br>Probe<br>o Have you seen or heard about any programs/activities? What do you think of those?<br>o If yes, how did you learn about the service/ programs?

	○ Any incident where the safety regulations have come helpful to the people?<br>○ Have you seen or received any material in relation to these?<br><br>• What can be done to reduce drowning and some of the practices that put people at risk of drowning?<br>• What sort of resources and facilities do you think are needed in your community for reducing the burden of drowning?
**Exit question**	During our conversation, you mentioned X, Y, Z measures that could be taken to reduce drowning in your community.<br>If you were to rank these from 1 to 5 (1 being most helpful and 5 least helpful for your household/community), what would the order be?<br><br>Does anyone have any other questions or anything you would like to say or discuss further in this group?
**Thank you**	
