# Supplementary material for: Community risk perceptions and recommendations for drowning prevention: A qualitative study across West Bengal, India
Source: PLOS Glob Public Health. 2026 Sep 24;6(9):e0007377. doi: 10.1371/journal.pgph.0007377 (PMC13606942; doi:10.1371/journal.pgph.0007377)
Supplement: Supplementary file 3 [file pgph.0007377.s003.pdf]

S1 Table: District-wise Demographic Characteristics of Respondents

District	Age-range	Total Respondents	Gender distribution (Female-F; Male-M)
Alipurduar	21-60	15	F-12, M-3
Birbhum	18-70	13	F-12, M-1
Coochbehar	31-55	14	F-11, M-3
Darjeeling	26-54	14	F-7, M-7
Hoogly	19-68	15	M- 8, F-7
Howrah	25-45	14	M-14
Jalpaiguri	25-66	16	F-12, M-4
Jhargram	18-56	14	F-7, M-7
Kalimpong	28-66	12	F-8, M-4
Malda	18-60	18	F-16, M-2
Murshidabad	19-60	13	F-8, M-5
N. Dinajpur	18-58	17	F-12, M-5
Nadia	27-70	13	F-10, M-3
Paschim Medinipur	30-62	14	F-3, M-11
Purba Barddhaman	24-60	12	F-1, M-11
Purba Medinipur	19-55	12	F-6, M-6
Purulia	19-64	20	F-15, M-5
S Dinajpur	21-52	11	F-10, M-1
S 24 Parganas	20-61	28	F-28
Bankura	24-65	14	F-5, M-9
Kolkata	27-60	13	F-13
N 24 Parganas	18-78	12	F-8, M-4
Paschim Barddhaman	25-58	15	F-14, M-1
